# The Emerging Role of Community Pharmacists in Remote Patient Monitoring Services

**DOI:** 10.3390/pharmacy8030166

**Published:** 2020-09-06

**Authors:** Amina Abubakar, Jessica Sinclair

**Affiliations:** Rx Clinic Pharmacy, Charlotte, NC 28227, USA; amina@rxclinicpharmacy.com

**Keywords:** remote patient monitoring (RPM), chronic disease, digital medicine, community pharmacy services, innovations in community pharmacy practice

## Abstract

Remote physiologic monitoring (RPM) services involve the transmission of patient-collected physiologic data to the healthcare team. These data are then analyzed to determine what changes may be needed to enhance patient care. While pharmacists may not be recognized as billing providers through some payers, there are opportunities for pharmacist collaboration with providers to enhance patient access to RPM services. Community pharmacist services are traditionally tied to a product, but pharmacists are skilled in medication management, disease state evaluation, and patient counseling, which are skills that can contribute to an elevated RPM program.

## 1. Introduction

Recent trends in the healthcare industry have highlighted promising studies of the integration of telemonitoring services into practice. The results from these studies show that patients are becoming increasingly interested in playing a larger role in their health [1]. As patients are interconnected via mobile devices and additional technologies in other aspects of their life, the healthcare industry is leveraging this technology to engage patients and assist in the management of chronic disease states. Many processes in the healthcare industry highlight the need to include the patient at the center of care, including the Pharmacists’ Patient Care Process outlined by the Joint Commission of Pharmacy Practitioners. This process highlights the cyclical nature of patient care, starting with collecting pertinent patient information, assessing and analyzing the collected information, developing and implementing a plan in collaboration with the patient, and following-up on key metrics [2]. By allowing the patient to take a more active role in their care, they may positively associate this model with satisfaction of care, and this may contribute to improved outcomes [3].

In a study conducted by Walker et al., four key themes were identified for the patient experience with telemonitoring services. Patients reported an increase in knowledge of the management of their chronic disease states in addition to feeling that their readings triggered actions by their healthcare providers. Patients also reported feeling a sense of reassurance and security, knowing that the results of their telemonitoring were being reviewed by their healthcare team. On the other hand, patients reported concerns of the additional burden of monitoring and the fear that the service might jeopardize their relationship with their providers [4]. These findings support the need to further explore the implementation of telemonitoring in practice.

There are many aspects of telemonitoring services that are yet to be studied in detail but these services provide a unique solution to address the growing costs of healthcare in the United States. Between the years of 2019 and 2028, the National Health Expenditure is projected to increase at an average annual rate of 5.4 percent, reaching an estimate of USD 6.2 trillion in 2028. Among the major payers in the United States, Medicare spending is expected to achieve the fastest rate of growth, and chronic conditions significantly contribute to this growing cost [5]. The economic burden of chronic conditions and mental health conditions are estimated to account for 90% of healthcare expenditures in the United States [6]. Studies have shown a positive impact of remote patient monitoring services on reducing hospitalizations and meeting key goals for management of major chronic disease states [7,8].

With the growing need to address healthcare expenditures, many have utilized team-based care as an approach to address gaps in therapy. Pharmacist integration into primary care practices has been associated with a positive impact on improved disease state control. Pharmacists, as the medication experts, bring a unique perspective to chronic disease state management. Common interventions made by pharmacists have included lifestyle counseling, medication reconciliation, addressing medication adherence, and recommendations for drug therapy problems [9,10]. A study also found a positive impact of pharmacist-led clinical services on physician quality metrics [11]. While studies have highlighted the value that the pharmacist can bring to a multidisciplinary care team, few studies have assessed the involvement of community pharmacists in telemonitoring services. Telemonitoring services present community pharmacists with a unique opportunity to impact the care of their patients, beyond a dispensed product.

## 2. Background

Approximately 67% of Medicare beneficiaries have two or more chronic conditions. Beyond this, these patients account for 94% of Medicare spending [12]. It is evident that chronic conditions have a significant impact on healthcare spending as well 30-day hospital readmissions. Due to this burden, Centers for Medicare and Medicaid Services (CMS) continues to develop new programs to mitigate spending. In 2015, chronic care management (CCM) was developed as a non-face-to-face service to address the management of patients with two or more chronic disease states. As a requirement for this service, patients must have a patient care plan that is reviewed and continually updated by the health care team [13]. CMS designed this program to address the complexities of managing patients with multiple comorbidities and devised plans to continue to expand upon other non-face-to-face services.

In 2018, CMS finalized the separate payment for the Current Procedural Terminology (CPT) code 99091, a remote patient monitoring code. They further specified that this code was payable with other services, such as CCM [14]. Starting in 2019, the remote patient monitoring service was expanded to three additional cremote physiologic monitoring (RPM) CPT codes, as outlined in Table 1 [15,16]. Continual expansion was observed when CMS announced a revision to the program in 2020. Another CPT code was added to account for additional increments of 20 min of time contributed by clinical staff within a calendar month. The supervision requirement for RPM services was modified from direct supervision to general supervision [17]. Per CMS, this service is now allowable under the supervising provider’s oversight but does not require the physical presence of the provider during the provision of the service. CMS approved this change to general supervision as RPM services were officially included as designated care management services, which can be provided under general supervision [18].

RPM is a service that provides clinicians with the digital data to develop and implement more informed treatment plans for patients enrolled in the service. It involves the collection, analysis and assessment, and interpretation of this digitally collected physiologic data [17]. The RPM service includes a CPT code for the setup of devices, 99453. This code is billable for the setup and education of appropriate device use with the patient or caregiver. The CPT code 99454 is utilized for supplying the device to collect the digital physiologic data. The remaining two codes outlined in Table 1, 99457 and 99458, are billable with the first and subsequent increments of 20 min of clinical staff time dedicated to the provision of RPM services within a calendar month. These codes specify that interactive communication with the patient or caregiver is a requirement of this service [18]. The CPT code 99091 specifically describes time accumulated by a physician or other qualified healthcare professional within a calendar month [17].

While CMS does not recognize pharmacists as qualified healthcare professionals and pharmacists cannot directly bill for RPM services, there are opportunities for pharmacist collaboration with primary care providers and other specialists to expand patient access to care through RPM. CMS authorizes pharmacists to work in collaboration with providers and offer services as employees or contracted personnel [17]. This such model has been implemented in different pharmacy practice settings [11,19,20]. With the transition of RPM services from direct to general supervision, community pharmacists working in tandem with qualified healthcare professionals can provide RPM services and collaborate in the collection, analysis, and interpretation of digitally transmitted physiologic data for the purposes of improving the care of patients that contribute to high Medicare spending, specifically high-risk patients with multiple chronic disease states.

Community pharmacies often provide services beyond dispensing, including but not limited to delivery services, medication therapy management, disease state screening, disease state prevention through counseling, and immunization services [21]. Through the provision of these patient care services and the counseling that accompanies medication dispensing, community pharmacists have developed trusting relationships with their patients. By leveraging the experience of community pharmacists as well as the relationship between the pharmacy and the patient, community pharmacists are well-positioned to expand into collaborative clinical services. RPM poses a unique opportunity for community pharmacists to begin to interact with patients beyond their four walls and utilize their expertise to triage incoming physiologic data for patients overseen by their supervising providers.

## 3. Implementation within the Community Pharmacy Setting

Rx Clinic Pharmacy is an independent community pharmacy located in Charlotte, North Carolina. The pharmacy offers clinical services in both the community and collaborative settings with collaborations extending from Charlotte, North Carolina to Florence, South Carolina. Clinical contracts have been established for pharmacists of Rx Clinic Pharmacy to work as contracted personnel with supervising providers. Starting in 2020, RPM services were extended to the community pharmacy setting as general supervision rules were approved by CMS. In April 2020, an RPM pilot of a total of 100 patients was launched in four collaborating clinics to monitor both blood pressure and weight utilizing devices equipped with cellular connectivity. Pharmacists, pharmacy technicians, and student pharmacists participated in this pilot program.

Prior to implementation of the program, the pharmacy assessed various platforms for the provision of RPM services. This included demos with HIPAA-compliant platforms that connect with devices to collect digital physiologic data. Cost-efficacy, ease of use, connectivity, and device options were all considered when selecting the most appropriate platform for use for the pilot program. Additionally, it was important for both the pharmacy and the providers to have access to this platform to review and update the care. The selected platform provided a dashboard for easily accessible information to track overall progress for the program. The platform also interfaced with cellular devices as well as devices with wireless connectivity with the Internet. For the purposes of this pilot program, cellular devices were selected for ease of use for the targeted patient population. Devices used for the program were tested by the manufacturer to meet the accuracy requirements and standards of the Food and Drug Administration (FDA) for blood pressure measurement devices and scales.

**Figure 1:**
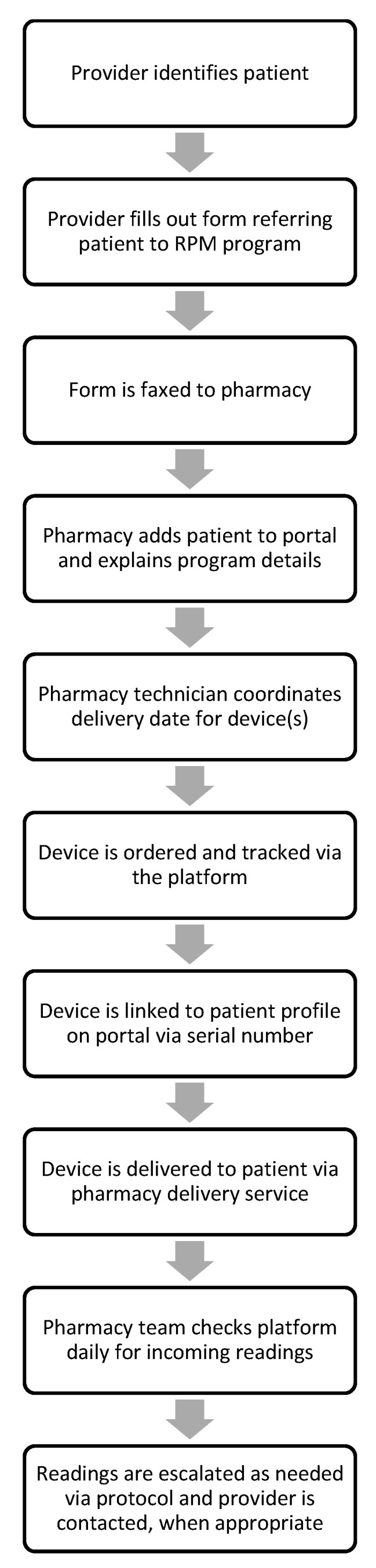
Remote physiologic monitoring (RPM) workflow within a community pharmacy.

The collaborating providers identified eligible patients who would qualify for and benefit from the RPM program. Verbal consent was obtained from these patients to enroll in the program. Once identified, the providers filled out a referral form for these patients as outlined in Figure 1 and this form was faxed to the pharmacy with supporting documentation. The referral form included essential patient information as well as device needs for the patient, either a scale or a blood pressure monitor. These devices were selected for the pilot program due to the cellular connectivity with the platform and for the ease of use in implementation. Numerous patients that were identified for the pilot program did not have wireless Internet and others reported concerns with the process of connecting devices to their wireless network. Once the referral form was received, the pharmacist then reviewed this information and contacted the patient by phone to explain more about the structure, purpose, and expectations for the program. Devices were ordered for the patients, and once the devices were received, the serial number of the device was connected to the patient profile on the platform by a pharmacy technician. This process defined the specific device assigned to the patient.

Rx Clinic Pharmacy leveraged current services to deliver the devices to the patients’ homes. Initial training and education were conducted by the pharmacist. Patients were educated on appropriate device use as well as methods for obtaining more appropriate readings. Once this process was complete, the patients were authorized to begin taking readings, and patients were encouraged to monitor daily, if not more frequently. Patients were instructed to take readings first thing in the morning for both blood pressure and weight readings. If a provider requested more frequent monitoring, patients were instructed to follow the monitoring plan outlined by their providers. Furthermore, patients could elect to take readings more frequently than once daily, if desired.

The pharmacy team, including pharmacists, pharmacy technicians, and student pharmacists, monitored incoming readings. If readings were outside of the defined range of normal, a pharmacy technician tasked the pharmacist to conduct patient outreach. The pharmacist contacted the patient by phone to determine if escalation was needed. This escalation was outlined in the protocol agreed upon between the pharmacy and the supervising provider. While some readings outside of the normal range could be addressed through patient counseling, others required escalation to the supervising provider. The pharmacy contacted the provider’s office to determine if the patient qualified for a telehealth or in-office visit. This process of RPM monitoring encouraged proactive communication with patients and between members of the healthcare team.

## 4. Challenges

One of the primary challenges of the RPM pilot was the difficulty for certain patients to consistently obtain readings. Some patients were diligent in taking readings to better understand their disease state control and help reach their clinical goals, but others found it troublesome to monitor. Additionally, the pharmacy team encountered communication challenges when reaching out to the patients by phone. Oftentimes, they had to make numerous phone call attempts, leave voicemails, and send letters reminding patients of the expectations for the RPM program. Considerations were made that these patients may have feelings consistent with the results outlined by the findings of Walker et al. that additional monitoring can be burdensome for patients [4]. Limited readings were obtained from these patients.

Additionally, a couple of patients reported challenges with data transmission. After troubleshooting technical issues with the platform support team as well as with the pharmacy, it was identified that the patients were taking readings appropriately, but the data were not being transmitted correctly. These patients were supplied with new devices to ensure appropriate collection of physiologic data, and the connectivity issues were resolved. Considerations for connectivity must be considered for devices, whether transmissions are received via a cellular connection or a wireless Internet connection. Data flow has proven to be a consistent concern in remote patient monitoring [22]. While cellular devices may simplify the process of device set-up, they do not completely resolve the complexities with troubleshooting connectivity issues.

There is a potential for human error when considering the digital collection of physiologic data. During the pilot, erroneous data were obtained when patients shared their devices with other individuals. Since each device was linked to a specific patient profile, these data were collected along with other readings. This was evident when monitoring weight changes where readings were greater than 50 pounds apart within a 24 h period. This type of information led to reinforcing the patient expectation that devices will only be used for the specific patient enrolled in the program. To address this issue, the pharmacy created a guide for troubleshooting issues with patient monitoring. This guide outlined a predefined set of questions to address readings that were outside of the normal range and to escalate the case as needed to the pharmacist or to the provider.

Another factor of human error relates to the quality of data obtained from the patients. For example, some patients obtained readings that were deemed inappropriate by the clinical staff monitoring the readings. It was determined that poor monitoring techniques contributed to this human error. Some patients reported placing their scales on carpet, rather than a hard surface, and others reported obtaining blood pressure readings that did not follow the recommendations for obtaining appropriate readings per the guidelines. The pharmacy team addressed these components as part of their counseling and interactive contact with the patients each month per the CMS requirements for RPM. After identifying the continued issues with monitoring errors, the pharmacy team created a step-by-step guide for patients to review monitoring instructions. This was provided to new patients at the time of initiation. Reinforcement of monitoring instructions was conducted by either the pharmacist or the pharmacy technicians and student pharmacists.

## 5. Successes

The results from each clinic were evaluated separately as well as collectively to determine overall successes for the RPM program. Between the period of April to June 2020, the frequency of monitoring increased across the four clinics and 100 patients. This highlighted the expansion of the program as well as the impact of education on appropriate monitoring. Some patients reported a positive experience with monitoring their readings and using it as immediate feedback to guide modifications in their lifestyle. By further evaluating the data, the pharmacy was able to identify key factors that might be impacting the data. This encouraged counseling patients on monitoring techniques.

Another positive impact of the RPM pilot was the increase in the number of referrals made to the clinic for telehealth or in-office visits. If during the RPM daily monitoring, it was identified that a patient would benefit from being seen by a provider for a medication evaluation and visit, the pharmacy contacted the clinic to assist in scheduling an appointment. This proactive approach led to improved provider oversight and retention in care, allowing providers to work more closely with their patients, which is critically important for patients who are high-risk and have multiple chronic conditions. Moreover, several of these visits led to medication changes and referrals to specialists. Providers had access to additional data through RPM to make informed decisions in the patient’s care. From the patient perspective, patients may feel a sense of security, knowing that their health care team is monitoring them in-between office visits [4].

This collaboration expanded clinical service offerings within the clinics as RPM services were implemented as new programs during this pilot. Additionally, this collaboration increased the frequency of interventions recommended by the pharmacy team. By partnering RPM services with other non-face-to-face services, such as CCM, the healthcare team built a program that encompassed intensive medication evaluation and management, lifestyle counseling, preventive screening recommendations, and enhanced coordination of care. Furthermore, this program supported the new role for a clinical pharmacist as a remote care manager. Through programs much like this pilot, pharmacists have an invaluable opportunity to impact patient care and provide value to medical providers and patients while working remotely.

## 6. Future Directions

In the current landscape, there is potential for continued rapid growth of RPM services. With increasing opportunities for telehealth and telemonitoring programs, there may be expansion of similar programs with other payers. In April 2020, North Carolina Medicaid announced the temporary addition of coverage for RPM services and aligned their program with the CPT codes outlined by Medicare. Although it was noted that the program and policies allow for temporary flexibilities, there is a demand for improved connectivity between providers and patients that may serve as an impetus for more permanent solutions [23]. RPM and telehealth services may contribute to decreased hospitalizations, improved care coordination, and proactive disease state assessment for patients that contribute to high spending in the healthcare system.

Advancements in technology have the potential to broaden the range of devices that are available for RPM services. Furthermore, these advancements may contribute to more reliable devices and higher quality data. While human error will continue to impact readings, there may be ways to minimize these variabilities in the future. Evaluation of data loss, connectivity factors, and other technical concerns will be vital to ensure that patients have positive feelings towards RPM programs. Beyond the devices, this pilot explored factors of the patient experience with RPM services, and it is evident that setting clear expectations at the time of enrollment and continual reinforcement throughout the program may lead to improved adherence with monitoring. Additional evaluation of patient qualifications may be necessary to identify the most appropriate patients for RPM programs.

There are many considerations for clinical services within the community pharmacy setting. While providers have traditionally visualized pharmacists in the dispensing role, services such as RPM pose an opportunity for expansion into cognitive community pharmacy services. Pharmacists have the skills and training to be effectively integrated into the provision of RPM. Their knowledge of medications, disease state evaluation, and counseling skills enhance the value within a healthcare team. Further studies are needed to assess the impact of community pharmacists on clinical and patient-driven goals outlined through RPM programs. Through this team-based approach to care, services such as RPM may serve as a gateway to strengthening community pharmacy partnerships with providers while also linking patients to patient care services in between office visits.

## Figures and Tables

**Table 1:** Remote physiologic monitoring services payable through Medicare.

Description	Code	Non-Facility Price *	Non-Facility Limiting Charge *	Facility Price *	Facility Limiting Charge *
Remote monitoring physiologic parameters, setup	99453	$18.77	$20.50	$18.77	NA
Remote monitoring physiologic parameters, device	99454	$62.44	$68.21	$62.44	NA
Remote monitoring physiologic parameters, 1st 20 min	99457	$51.61	$56.38	$32.84	$35.88
Remote monitoring physiologic parameters, each additional 20 min	99458	$42.22	$46.13	$32.84	$35.88

* National payment amount in US Dollars.
